# Use of remote data collection methodology to test for an illusory effect on visually guided cursor movements

**DOI:** 10.3389/fpsyg.2022.922381

**Published:** 2022-09-02

**Authors:** Ryan W. Langridge, Jonathan J. Marotta

**Affiliations:** Perception and Action Lab, Department of Psychology, University of Manitoba, Winnipeg, MB, Canada

**Keywords:** Ebbinghaus illusion, Titchener circles, cursor control, perception, action

## Abstract

Investigating the influence of perception on the control of visually guided action typically involves controlled experimentation within the laboratory setting. When appropriate, however, behavioral research of this nature may benefit from the use of methods that allow for remote data collection outside of the lab. This study tested the feasibility of using remote data collection methods to explore the influence of perceived target size on visually guided cursor movements using the Ebbinghaus illusion. Participants completed the experiment remotely, using the trackpad of their personal laptop computers. The task required participants to click on a single circular target presented at either the left or right side of their screen as quickly and accurately as possible (Experiment 1), or to emphasize speed (Experiment 2) or accuracy (Experiment 3). On each trial the target was either surrounded by small or large context circles, or no context circles. Participants’ judgments of the targets’ perceived size were influenced by the illusion, however, the illusion failed to produce differences in click-point accuracy or movement time. Interestingly, the illusion appeared to affect participants’ movement of the cursor toward the target; more directional changes were made when clicking the Perceived Large version of the illusion compared to the Perceived Small version. These results suggest the planning of the cursor movement may have been influenced by the illusion, while later stages of the movement were not, and cursor movements directed toward targets perceived as smaller required less correction compared to targets perceived as larger.

## Introduction

The Ebbinghaus illusion, also referred to as the Titchener circles illusion, is a well-known size-contrast illusion in which the perceived size of a central target circle is made to appear smaller or larger than its true size when surrounded by a ring of larger or smaller context circles, respectively. The strength of the illusion can be manipulated by altering the size and distance of the context circles relative to the target circle; smaller distances between the target circle and the surrounding annulus increase the perceived size of the target circle, while larger distances decrease its perceived size (Massaro and Anderson, 1971; Roberts et al., 2005; Knol et al., 2015). Visual illusions such as the Ebbinghaus illusion provide an opportunity to explore the degree of separation between a visual system dedicated specifically to the processing of a stimulus’ perceptual properties, and a visual system dedicated specifically to the execution of visually guided action toward that stimulus. A functional separation of these two visual systems, as proposed by Goodale and Milner (1992) and Milner and Goodale (2006) suggests that a size-contrast illusion such as the Ebbinghaus illusion should primarily influence one’s perceptual judgments of a stimulus’ size processed within the ventral stream, while the visually guided action toward that stimulus, guided by computations performed by the dorsal stream, should be largely unaffected by the illusory context.

Traditionally, research of this nature involves measuring participants’ grip aperture when reaching to grasp a circular disk embedded within the illusion and drawing a comparison to participants’ judgments of the disk’s perceived size in response to the illusory context. A number of studies have provided evidence supporting the theory that visually guided grasping actions are immune to the influence of the Ebbinghaus illusion by describing relatively stable, non-changing grip apertures in comparison to perceptual size-judgments that vary as a function of the illusory context (Aglioti et al., 1995; Haffenden and Goodale, 1998; Marotta et al., 1998; Danckert et al., 2002). Yet others have provided contradictory evidence suggesting that both perceptual judgements and visually guided action are influenced to some degree by visual illusions (Pavani et al., 1999; Franz et al., 2000; Franz and Gegenfurtner, 2008). Some of these contradictions may result from variations in the presentation of the illusion across investigations (e.g., differing size and presentation of the targets, variable number of surrounding context circles, etc.), as well as variation in the methods used to measure the perceptual influence of the illusion itself: for instance, having the participants adjust the size of a comparison stimulus to match the size of the target stimulus (e.g., Aglioti et al., 1995; Haffenden and Goodale, 1998) vs. using the distance between the index finger and thumb during perceptual estimation of the stimulus size (e.g., Haffenden et al., 2001). Incongruencies in study design such as these may explain why some studies have demonstrated an effect of the Ebbinghaus illusion on visually guided action, while others have not.

Some who argue for an “illusion immunity” of visually guided action contend that the apparent effects of the illusion on grip aperture may be the result of an obstacle avoidance mechanism, suggesting any observed changes in grip aperture are caused by the proximity of the context circles to the target circle, rather than in response to a perceived change in target circle size. Certain studies have found evidence for this hypothesis (Haffenden and Goodale, 2000; Haffenden et al., 2001; De Grave et al., 2005; Gilster et al., 2006). However, there are others who have provided evidence suggesting the positioning of the context circles is not a sufficient explanation for the observed changes in grip aperture, and therefore these changes must arise in response to a change in the perceived size of the target circle (Franz et al., 2003; Franz and Gegenfurtner, 2008; Kopiske et al., 2016).

The extent to which the Ebbinghaus illusion influences the precision and timing of other visually guided actions such as pointing or tapping has also been explored (van Donkelaar, 1999; Handlovsky et al., 2004; Alphonsa et al., 2016; Knol et al., 2017). Instead of requiring an appropriately scaled grasp matched to the boundaries of a circular target object, these actions require performing an accurate movement toward the target’s center, and the influence of the illusion is typically investigated by testing for the presence of a speed-accuracy trade-off (e.g., Fitts’ Law; Fitts, 1954), when acting on targets perceived to be smaller or larger than their veridical size. However, as with investigations of grasping the Ebbinghaus illusion, the results of studies involving this type of action are also often contradictory. For example, van Donkelaar (1999) originally demonstrated slower movement times toward targets perceived to be smaller, however, attempts to replicate these results failed to demonstrate an influence of the illusion on movement time (Fischer, 2001). A critical difference between the early van Donkelaar (1999) and later Fischer (2001) studies involved visibility of the hand: participants in van Donkelaar’s original study did not have visual feedback of their hand, while those in Fischer’s later study were able to see their hand while performing the task. In a similar study, Alphonsa et al. (2016) adapted a typical Fitts tapping task to include qualities of both the Ebbinghaus and the Muller-Lyer illusions. Despite the physical size and distance of the two target stimuli being identical, participants were more accurate when tapping targets in the “illusory easy” condition, in which the combination of illusions increased the perceived size of the target. However, the effect of the illusion on participants’ tapping accuracy was only observed during “discrete” tapping, where visual feedback of the target was removed. Together, these results suggest that the degree to which the illusion influences movement time may be related to the amount of visual information available. In instances where an influence of the Ebbinghaus illusion is observed, the evidence appears to suggest increased movement time for actions directed toward stimuli perceived to be smaller than their veridical size.

More than ever before, the research community is now looking for ways in which to adapt classic in-person data collection procedures into formats that allow participant data to be collected remotely. While research utilizing the use of online questionnaires and other forms of self-report measures has long benefited from remote data collection, behavioral research requiring the increased experimental control provided by the laboratory setting, as well as the use of expensive, often cumbersome equipment, has necessitated these types of experiments to be conducted in the lab, under experimenter supervision. The aim of this study was to test the implementation of remote data collection methods to investigate behaviors such as visually guided movement. In particular, this study measured the extent to which perceptual information influences participants’ cursor movements when using a laptop trackpad to perform a point-and-click task, an increasingly common behavior for many people. Despite being far removed from the type of action humans’ visual system evolved to facilitate, this type of task provides an interesting context in which to measure the visual perception-action relationship. Onscreen cursor movement requires a transformation from egocentrically defined finger movements—typically controlled via the vision-for-action dorsal stream—into the on-screen environment, where allocentric references are critical for the guidance of the cursor to the desired location and are therefore likely influenced to some degree by the vision-for-perception ventral stream (Goodale and Milner, 1992; Milner and Goodale, 2006). Cursor movements have also been shown to adhere to Fitts’ Law (Sutter et al., 2011), suggesting there is a speed-accuracy trade off present following this type of visuomotor transformation as well. While the processes involved in reaching toward and grasping a physical object are inherently different from those which serve interaction with 2-D stimuli (Freud et al., 2018; Ozana et al., 2020), the use of a trackpad to control an onscreen cursor has become an increasingly prevalent behavior for many individuals. As such, exploring the ways in which the visual system performs these transformations is an interesting direction of study.

If the transformation from finger movement into cursor movement requires some degree of perceptual control, we may expect one’s cursor movements to be influenced by their perception of the onscreen stimuli being clicked on. Using the Ebbinghaus illusion to influence participants’ perceptions of target size, we tested the degree to which participants’ cursor movements toward targets embedded within the Ebbinghaus illusion were influenced by the illusory context. In doing so, we incorporated features of a classic behavioral investigation into an experiment that could be conducted remotely, using the participants’ own laptop device. The results of three experiments are detailed here, along with a discussion of the potential strengths and limitations present when conducting this type of experiment remotely.

## Methods

### Participants

All participants were recruited through the Psychology Department Undergraduate Participant Pool at the University of Manitoba and participated in exchange for course credit toward their Introduction to Psychology course (Table 1). All participants self-reported having either normal or corrected to normal vision (e.g., wearing glasses, contact lenses, corrective eye-surgery, etc.), and were right-hand dominant, as determined by a modified version of the Edinburgh Handedness Inventory (Oldfield, 1971). All participants also self-reported using their right hand to control the cursor when using a computer. All participants provided informed consent prior to participation, and all procedures were approved by the Psychology/Sociology Research Ethics Board (PSREB) at the University of Manitoba.

**TABLE 1:** Demographic information and experimental instructions.

	Demographic information	Instructions
Experiment 1	*N* = 50 (41 female, 9 male), ages 18–32 years (*M* = 20.10, *SD* = 3.38)	“Press the Continue button below to proceed to the next set of trials”
Experiment 2	*N* = 50 (38 female, 12 male), ages 18–44 years (*M* = 20.02, *SD* = 4.40)	“Try to be faster when clicking! Remember: The goal is to click the center of the target circle AS QUICKLY AS POSSIBLE”
Experiment 3	*N* = 50 (38 female, 11 male, 1 undeclared), ages 17–23 (*M* = 18.96, *SD* = 1.59)	“Try to be more accurate when clicking! Remember: The goal is to click the CENTER of the target circle AS ACCURATELY AS POSSIBLE.”

### Experiment construction

The experiment was built using lab.js (Henninger et al., 2021) a free online study builder designed for the behavioral and cognitive sciences.

### Cursor presentation

To ensure accuracy during performance and to avoid any positional biases in cursor position (Phillips et al., 2001, 2003), participants’ cursor was set to appear as a “crosshair,” rather than the default arrowhead pointer.

### Stimuli presentation

The sizes of the stimuli were measured in logical pixels (px), mapped accordingly to the physical pixels of the device’s screen based on the device’s screen resolution and device-pixel-ratio (DPR). Using logical pixels to design the on-screen stimuli meant the stimuli sizes remained relatively similar across devices; the DPR of devices with significantly higher screen resolutions prevented the stimuli from appearing drastically smaller than on devices with lower resolutions. Unless specified otherwise, the term “pixels” and the abbreviated “px” refers to logical pixels. Information regarding the DPR of the device being used to perform the experiment, as well as the monitor resolution (Table 2), size of window content, and size of the browser viewport was included in the metadata collected by lab.js each time a participant completed the experiment.

**TABLE 2:** Screen resolution (logical pixels) and device pixel ratio (DPR).

Experiment 1			Experiment 2			Experiment 3		
Screen resolution	Device pixel ratio (DPR)	Number of participants	Screen resolution	Device pixel ratio (DPR)	Number of participants	Screen resolution	Device pixel ratio (DPR)	Number of participants
1,280 × 720	1.5	6	1,280 × 720	1.5	2	1,280 × 720	1.5	5
1,280 × 800	2	1	1,280 × 800	1	1	1,280 × 800	1	2
1,366 × 768	1	9		2	3		2	2
1,368 × 912	2	1	1,366 × 768	1	12	1,366 × 768	1	9
1,440 × 900	1	5	1,368 × 912	2	1	1,440 × 900	1	8
	2	16	1,440 × 900	1	13		2	14
1,500 × 1,000	2	1		2	14	1,504 × 1,003	1.5	1
1,504 × 1,003	1.5	2	1,536 × 864	1.25	1	1,536 × 864	1.25	6
1,536 × 864	1.25	8	1,600 × 900	1	1	1,792 × 1,120	2	1
1,680 × 1,050	1	1	1,792 × 1,120	2	2	1,920 × 1,080	1	1
						1,920 × 1,200	2	1

The different target types used in this experiment are presented in Table 3. The stimuli were presented within an 800 × 600 px container, so they could be viewed on a wide range of screen sizes and resolutions. Targets appeared as white circles against a black background, and were presented either alone (Control targets), or surrounded by an annulus of context circles. The size and position of these context circles determined the direction of the illusion. In addition to the traditional variations of the Ebbinghaus illusion (small context circles positioned close to the target vs. large context circles positioned far from the target) a Perceived Large (Far) target was also included (small context circles positioned far from the target), in an attempt to observe the effect of the illusion while controlling for the context circles’ proximity to the target.

**TABLE 3:** Target type and dimensions.

Target type	Target circle diameter (px)	Context circle diameter (px; proportion of target circle diameter)	Distance from edge of target circle to inner edge of context circle (px)
Control (Small) 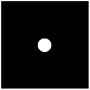	60	–	–
Control (Regular) 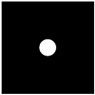	70	–	–
Control (Large) 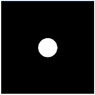	80	–	–
Perceived Small 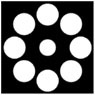	70	96 (1.37)	58
Perceived Large 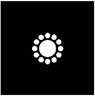	70	27 (0.39)	11
Perceived Large 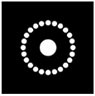 (Far)	70	27 (0.39)	73

When presented on a 13-inch screen at a resolution of 3000 × 2000 physical pixels and a device pixel ratio = 2, the diameter of the Control Small target measured approximately 11 mm, the diameter of the regular sized targets measured approximately 13 mm, and the diameter of the Control Large target measured approximately 15 mm.

### Experiment hosting

Lab.js supports several options for online study deployment, as well as exportation for offline data collection. The current online study was hosted on Github^1^ (Github, 2021), an online, open-source software development platform that provides internet hosting. Participants were provided with the link to the experiment through the University of Manitoba’s online external study management system. As GitHub does not support the saving of participant data, participant data was saved to a secure, realtime database using Google Firebase^2^ (Firebase, 2021) and exported using custom programming in R (R Core Team., 2020)

### Procedure

#### Self-report

Once directed to the experiment website, participants were asked to confirm their use of the touchpad/trackpad of a laptop computer to complete the experiment (use of a physical mouse or touchscreen device to control the on-screen cursor was not permitted). Participants were then presented with a consent form and were required to provide consent before continuing. Next, participants reported to the best of their knowledge the type of device they were using to complete the experiment, as well as the device’s screen size, and were asked to confirm once again that they were using their finger on the device’s touchpad/trackpad rather than a physical mouse or touchscreen device. Participants then provided demographic information regarding their vision (e.g., normal or corrected-to-normal), sex assigned at birth, and handedness.

#### Screen set-up

The first task involved participants using their cursor to click on five circular targets (diameter = 2 px) presented in sequence on their computer screen, one target each positioned in the center of the screen, 200 px to the left and right of center (these positions corresponded to the position of the targets during the experimental trials), and 150 pixels above and below the screen’s center. The presentation of each target was preceded by a 200 ms mask to prevent any afterimages of the previous target. The recorded clicks at these target positions were used during analysis to confirm the metadata regarding the device’s screen size and resolution were accurate, as well as to use as a reference point for the target’s position during the experimental trials.

#### Instructions

Following the screen setup task, participants were presented with a set of instructions explaining the experiment, beginning by asking participants to maintain approximately 2 feet (“2 rulers distance”) between their head and the computer screen, in an attempt to maintain consistent viewing distance across participants (see Li et al., 2020 for additional options for controlling viewing distance). Next, participants were informed that a target circle would appear on the screen and were instructed to click on the center of the on-screen target “AS QUICKLY AND AS ACCURATELY AS POSSIBLE.” Example images were provided to help describe the task, and to distinguish the “target circle” from the surrounding context circles.

#### Experimental task

Each trial began with a gray start button (diameter = 30 px), presented 250 px below the center of the screen. Participants were required to click the start button to initiate each experimental trial, and each experimental trial was preceded by a 200 ms mask. Each trial consisted of a target presented either 200 px to the left or right of the screen’s center. Participants completed the trial by moving their cursor to the target and clicking within the target circle’s boundaries, after which the target would disappear, and the start button would reappear to begin the next trial. Only clicking within the target circle’s boundary ended the trial; clicks outside the boundaries were not recorded. There were no time constraints on the presentation of the stimuli, and the target remained on the screen until it was clicked.

Participants completed a set of 12 practice trials, during which each target type was presented twice, once on the left and once on the right side of the screen. Prior to the onset of the experimental trials, participants were once again reminded to “click the center of the target circle as quickly and accurately as possible.” Participants then completed 60 randomized experimental trials (each unique combination of target type and on-screen position shuffled without replacement, then re-shuffled), such that each target type appeared five times on the left side of the screen, and five times on the right. Participants were then given an opportunity to take a break and instructed to “Press the Continue button below to proceed to the next set of trials” before completing another 60 randomized experimental trials.

#### Perceptual comparisons

After completing the 120 experimental trials, participants completed a forced-choice perceptual size comparison task, in which they were presented with two different target types and were instructed to click which target they believed to be larger. These comparisons were included to check if the illusory context was effectively manipulating the perceived size of the targets. The two targets being compared were never the same type, and each target type was compared with the other five target types twice, appearing once on the left and once on the right side of the screen at positions corresponding to those used during the experimental trials (200 px to the left and right of center) equaling a total of 30 trials. Participants were allowed to take as much time as needed to make their selection. Participants whose responses were incorrect when comparing the three veridically different control targets (Control Small, Control Regular, and Control Large) were excluded from the analysis. After finishing the perceptual comparison task, all participant data was uploaded to the secure database, and participants were debriefed and directed to exit their browser.

#### Manipulation of task instructions

To test the influence of the experimental instructions on participants’ performance, and to explore if the particular demands of the task influenced the degree to which the illusory context affected participants’ cursor movements, three separate experiments were conducted. The experimental design and construction were identical for each experiment, with the exception of the message received by participants following the first block of 60 trials. Participants in Experiment 1 were instructed to simply begin the next set of trials, while participants in Experiments 2 and 3 were instructed to prioritize speed or accuracy, respectively, during the next set of trials (Table 1). To control for the varying range of devices used by participants, as well as the various screen sizes and resolutions, each experiment was analyzed separately as a within-subject repeated measure design.

### Dependent variables

Participants’ temporal and spatial cursor data were measured in lab.js using the Mousetrap plugin (Kieslich and Henninger, 2017). This movement data can be used to explore a wide variety of experimental variables tailored to the specific research question being investigated. In this study, movement variables that would typically be investigated in a traditional reach-to-grasp or reach-to-point study such as accuracy and duration of the movement, deceleration phase, as well as measures of the movement trajectory itself were explored. For each dependent variable, averages were calculated within each unique condition to create a mean condition value for each participant.

#### Click-point accuracy

The radial error, calculated as the Euclidian distance (px) between the target’s center and the location of the participant’s click point was used to provide an absolute value representing click-point accuracy. Smaller values indicate click-point positions closer to the target’s center and higher accuracy.

#### Movement time

The amount of time from the onset of cursor movement to the time at which participants clicked the target was measured in milliseconds (ms).

#### Deceleration phase

To test for an effect of the illusion on participants’ online control of the cursor, the phase of deceleration—defined as the proportion of the overall (100%) movement following the point in time at which peak velocity was reached—was analyzed.

#### Area under the curve

Cursor trajectories were spatially normalized using 101 equidistant points (i.e., 0–100% of the movement distance) along the original cursor trajectory using the mt_spatialize function in the mousetrap package. The Area under the curve (AUC) was defined as the geometric area (px) between the trajectory and an idealized (straight) path connecting the trajectory’s start and end positions. The R function polysimplify from the polyclip package (Johnson and Baddeley, 2019) was used to separate the cursor deviations from the idealized path and the polyarea function from the pracma package (Borchers, 2021) was used to combine the area of these deviations. Doing so produced an absolute deviation value by treating participants’ deviations as additive rather than subtractive (the default method in the mousetrap package). Larger AUC values represent greater deviation from the idealized path, and a more curved cursor trajectory.

#### Time of maximum deviation

The stage of movement—defined as a proportion of the overall (100%) movement—at which the cursor position deviated the farthest from the idealized path connecting the trajectory’s start and end positions.

#### Number of directional changes

Using the same spatially normalized trajectories mentioned above, the frequency at which participants changed the direction of their cursor movement in either the horizontal or vertical axes during their movement toward the target in each trial was counted and averaged to create a mean condition value for each participant.

### Data analysis

The cursor data collected by the Mousetrap plugin was analyzed using the Mousetrap package (Kieslich et al., 2019) in R (R Core Team., 2020). Each trial ended once participants clicked the target, and therefore the final x and y coordinates of these cursor trajectories were used to define the position of the participants’ click point on each trial. Each cursor trajectory was inspected manually, to ensure the cursor position was recorded effectively throughout the trial. Additionally, as the logging resolution (the intervals at which the cursor position was recorded throughout the movement) had the potential to vary across devices, the logging resolution of each dataset was checked using the mt_check_resolution function in the mousetrap package. In all cases, the logging resolution was deemed satisfactory.

Experimental trials involving the Perceived Large (Far) target were removed from the analysis (see first paragraph of the “Results” section below). Each dependent variable was therefore analyzed using a 2 (Time: Pre-Break vs. Post-Break) × 2 (Position: Left vs. Right) × 5 (Target Type: Control Small vs. Perceived Small vs. Control Regular vs. Perceived Large vs. Control Large) within-subjects repeated measures ANOVA. All statistical analyses were conducted using SPSS (version 23.0). A Greenhouse-Geisser correction was used to address any violations to sphericity. Violations to the assumption of normality were identified by inspecting the normality of the residual values produced by the repeated measures ANOVA. In cases where the residual values were significantly and consistently non-normal, a transformation was applied to correct the non-normal data. All analyses were conducted using alpha = 0.05, and Bonferroni adjusted *p*-values were applied to all *post-hoc* comparisons used to analyze any significant interactions.

## Results

In all three experiments, analysis of participants’ perceptual comparison scores consistently indicated that the Perceived Large (Far) target was not successful in inducing the desired increase in perceived target size (participants reported an increase in the target’s perceived size in as few as 32% and no more than 58% of comparisons). This is likely due to the increased distance between the context circles and the target circle. Proximity of the context circles to the target circle is known to play an important role in the direction and magnitude of the Ebbinghaus illusion’s effect, with closer context circles increasing the size of the target circle, and farther context circles minimizing the size of the target circle (Massaro and Anderson, 1971; Knol et al., 2015), as also occurs in the Delboeuf illusion (Roberts et al., 2005). Based on the lack of any useful effect of the illusion, experimental trials involving the Perceived Large (Far) target were not analyzed.

### Experiment 1

#### Excluded data

A coding error made it possible for participants to begin their cursor movements immediately after clicking the start button, during the 200 ms mask prior to presentation of the target. This meant that any cursor movement that was executed during the 200 ms mask was not captured as part of the experimental trial. In total, 1.84% of all trials involved uncaptured cursor movement during the 200 ms mask and were therefore excluded from analysis. An additional 0.10% of all trials were removed due to missing time timestamp data (the timepoints throughout the trial at which cursor position was captured). Trials lasting longer than 5,000 ms to perform the task were also removed. This cut-off was determined to be excessive based on inspection of participants’ movement time data during analysis and accounted for 0.16% of the total number of trials. Finally, while the onscreen target represented the only “clickable area” on the screen, this clickable area was defined using square boundaries, which meant that in rare cases, participants could in fact click “outside” the circular target, in the corners of the square boundary. This occurred in 0.02% of trials, all of which were excluded from analysis. In total, 2.12% of experimental trials were excluded from analysis in Experiment 1.

#### Perceptual comparisons

As the time spent comparing the onscreen targets may have influenced participants’ perceptual responses, the average trial durations for each perceptual comparison are provided for context in Table 4. Participants’ perceptual comparison scores are provided in Table 5. Participants’ responses followed the direction of the illusion with a generally high consistency: over 75%, except for the comparisons involving the Perceived Large (Far) target. Additionally, a small portion of participants reported the Perceived Small target as being smaller than the veridically smaller Control Small target (20% when the Perceived Small target was on the left side of the screen, and 22% when it was on the right side of the screen). When comparing the Perceived Large target on the right side of the screen with the Control Large target on the left side of the screen, 25% of participants reported the Perceived Large target as being larger, however, this pronounced effect of the illusion disappeared when the target positions were reversed (0% when the Perceived Large target was on the left and the Control Large target was on the right).

**TABLE 4:** Average perceptual comparison durations (ms).

Target position		Average duration (ms)		
Left	Right	Experiment 1	Experiment 2	Experiment 3
Control Small	Perceived Small	2513.83 (1499.19)	2251.92 (1975.41)	2737.29 (2196.80)
Control Small	Control Regular	1570.12 (611.02)	1387.04 (476.81)	1717.00 (654.22)
Control Small	Perceived Large (Far)	1925.68 (881.48)	1902.26 (1278.20)	2305.79 (1218.07)
Control Small	Perceived Large	1598.53 (519.55)	1618.43 (712.68)	1994.65 (948.93)
Control Small	Control Large	1412.72 (541.48)	1268.44 (401.80)	1573.73 (588.57)
Perceived Small	Control Small	2833.39 (1958.59)	2343.64 (1814.58)	3043.41 (1937.97)
Perceived Small	Control Regular	1827.62 (604.81)	1653.86 (629.30)	2424.98 (1856.95)
Perceived Small	Perceived Large (Far)	2609.35 (4426.98)	2467.01 (1563.89)	2473.24 (1268.47)
Perceived Small	Perceived Large	1908.66 (722.92)	2068.09 (1600.03)	2451.17 (1894.03)
Perceived Small	Control Large	1682.91 (519.62)	1561.28 (542.36)	1763.56 (811.28)
Control Regular	Control Small	1652.10 (470.85)	1387.32 (441.16)	1715.82 (600.22)
Control Regular	Perceived Small	2231.34 (1175.06)	2077.99 (1575.86)	2142.87 (928.95)
Control Regular	Perceived Large (Far)	3514.77 (6342.86)	2754.90 (4035.91)	2938.42 (1957.30)
Control Regular	Perceived Large	2197.54 (1705.32)	1886.28 (1089.75)	2381.06 (1510.89)
Control Regular	Control Large	1608.49 (510.66)	1481.97 (537.51)	1830.50 (1154.93)
Perceived Large (Far)	Control Small	2012.69 (1015.28)	1640.08 (802.74)	1861.31 (642.35)
Perceived Large (Far)	Perceived Small	2497.80 (2788.96)	2314.77 (2074.66)	2416.01 (1445.88)
Perceived Large (Far)	Control Regular	2478.00 (1622.94)	2918.56 (5145.41)	2813.16 (1978.17)
Perceived Large (Far)	Perceived Large	2267.54 (1174.96)	3072.69 (5504.96)	3148.43 (2219.74)
Perceived Large (Far)	Control Large	1694.13 (589.59)	1683.71 (967.56)	1840.67 (676.94)
Perceived Large	Control Small	1833.64 (607.44)	1542.29 (592.46)	1929.27 (794.80)
Perceived Large	Perceived Small	2097.17 (988.34)	1888.54 (982.18)	1996.51 (1172.08)
Perceived Large	Control Regular	2255.42 (1028.70)	2268.09 (2099.42)	2642.57 (1732.29)
Perceived Large	Perceived Large (Far)	2483.62 (1630.25)	2532.55 (3727.42)	3149.33 (2248.89)
Perceived Large	Control Large	2170.40 (1606.41)	1695.23 (759.18)	2131.64 (1061.51)
Control Large	Control Small	1533.86 (463.12)	1258.86 (394.20)	1579.30 (592.23)
Control Large	Perceived Small	1841.06 (968.74)	1531.24 (580.31)	1853.19 (604.94)
Control Large	Control Regular	1582.56 (570.45)	1380.62 (362.76)	1910.71 (1120.91)
Control Large	Perceived Large (Far)	1763.81 (626.15)	1652.93 (762.92)	1847.74 (718.73)
Control Large	Perceived Large	2349.27 (1549.00)	2198.07 (1378.20)	2325.61 (1051.54)

Standard deviations presented in parentheses.

**TABLE 5:** Experiment 1 perceptual comparisons.

Onscreen position		Right					
		Control Small	Perceived Small	Control	Perceived Large (Far)	Perceived Large	Control Large
**Left**	**Control Small**	–	80%*	–	90%	100%	–
	**Perceived Small**	78%*	–	**92%**	**92%**	**94%**	96%
	**Control**	–	**88%**	–	**44%**	**76%**	–
	**Perceived Large** (Far)	94%	**94%**	**32%**	–	**82%**	98%
	**Perceived Large**	100%	**96%**	**76%**	**84%**	–	100%
	**Control Large**	–	98%	–	94%	76%*	–

Scores represent the percent of comparisons that demonstrated the expected size ordering (Smallest to Largest): Control (Small) < Perceived Small < Control < Perceived Large (Far) < Perceived Large < Control Large. **Bolded scores represent the comparisons between the same-sized targets.** Comparisons between Control stimuli (all 100%) not included.

*The fact that this value is less than 100% suggests that on the remaining percent of trials participants reported the illusory target as smaller or larger than the veridically smaller (Control Small) or larger (Control Large) targets respectively, suggesting the presence of an exaggerated illusory effect.

#### Click-point accuracy

Examining the distributions of participants’ average accuracy scores within each condition indicated non-normal, moderately to severely positively skewed data in all conditions. To address this violation to normality, a log transformation was applied to the data. The data reported here have been back-transformed into their original units for ease of interpretation.

**FIGURE 1:**
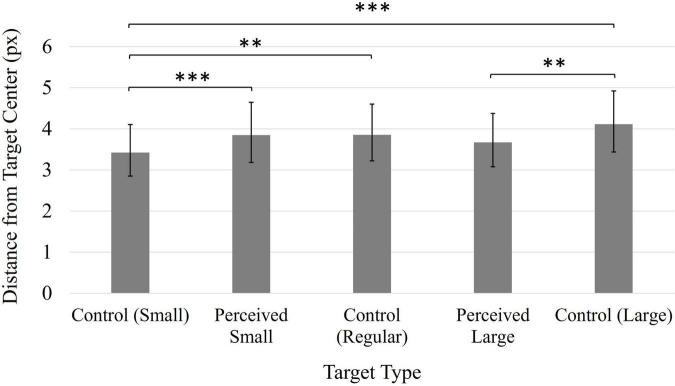
Average distance from click position to target center. Values have been back-transformed into original measurement value (px). Smaller values indicate higher accuracy. Error bars represent 95% confidence intervals. ^**^p < 0.01, ^***^p < 0.001.

A significant main effect of Time, *F*(1, 49) = 7.09, *p* < 0.05, η*_*p*_*^2^ = 0.13, indicated that participants were more accurate in their click positions during the first block of trials (*M* = 3.62 px, 95% CI [3.04, 4.31]) compared to the second block of trials (*M* = 3.94 px, 95% CI [3.27, 4.73]). A significant main effect of Target, *F*(4, 196) = 10.91, *p* < 0.001, η*_*p*_*^2^ = 0.18, indicated that participants were generally most accurate when clicking on the Control Small target (Figure 1). This is likely due to the Control Small target representing a smaller clickable area compared to the other targets, requiring participants to be more accurate overall. However, there were no significant comparisons amongst the three same-sized targets (Perceived Small, Perceived Large and Control Regular targets), suggesting the presence of the illusion did not influence participants’ clicking accuracy.

#### Movement time

**FIGURE 2:**
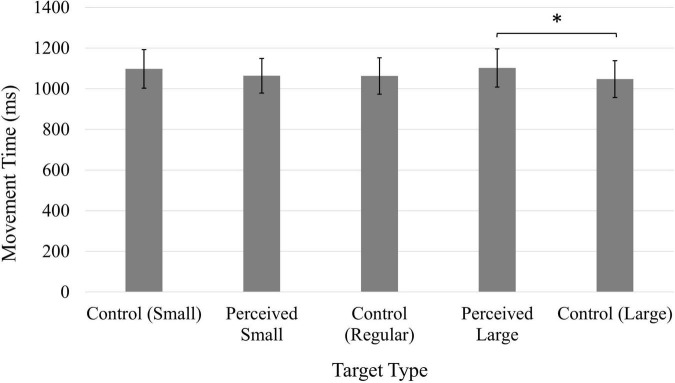
Average movement time (ms). Error bars represent 95% confidence intervals. *p < 0.05.

A significant main effect of Target, *F*(4, 196) = 4.87, *p* < 0.01, η*_*p*_*^2^ = 0.09, suggested the type of target influenced participants’ speed when performing the task (Figure 2). However, the only significant comparison was between the Perceived Large and the Control Large targets; movement time was significantly longer when clicking on the Perceived Large target.

#### Deceleration phase

The distributions of the average deceleration phase lengths were determined to be non-normal (moderately to severely negatively skewed). A square transformation was applied to the data to correct this violation of normality. The data reported here have been back-transformed into their original units for ease of interpretation.

Participants’ deceleration phases were not affected by Time, Target Type, or Position (all *ps* > 0.05). The average deceleration period across all conditions was 87.28% (*SD* = 0.62%) of the total movement.

#### Area under the curve

**FIGURE 3:**
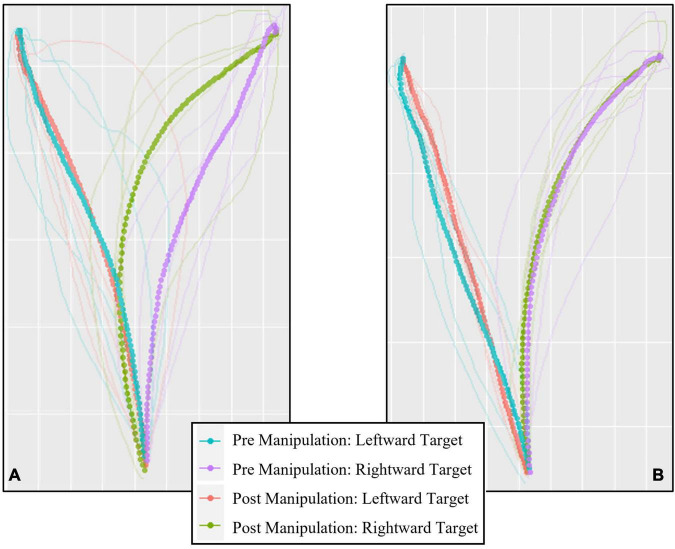
An example of the trajectories generated by a single participant executing cursor movements toward the Perceived Large **(A)** and Perceived Small **(B)** target. Solid lines represent data from each trial, dotted lines represent the average trajectory for each condition. Plot generated using the mt_plot_aggregate function from the mousetrap package in R (Kieslich and Henninger, 2017).

Figure 3 presents an example of the cursor trajectories executed by one participant when clicking on the Perceived Large (Figure 3A) and Perceived Small target (Figure 3B). A significant main effect of Position, *F*(1, 49) = 7.01, *p* < 0.05, η*_*p*_*^2^ = 0.13, indicated that participants executed more curved cursor movements when the target was presented on the right side of the screen (*M* = 17309.72 px, 95% CI [15342.79, 19276.64]) compared to when presented on the left (*M* = 14608.33 px, 95% CI [12790.36, 16426.31]).

#### Time of maximum deviation

A main effect of Target was found to be significant, *F*(4, 196) = 3.331, *p* < 0.05, η*_*p*_*^2^ = 0.06, and a general trend suggested the maximum deviation occurred earliest when clicking on the Perceived Large target (*M* = 17.83% of the movement, 95% CI [16.18, 19.49]), followed by the Control Regular (*M* = 18.08%, 95% CI [16.56, 19.60]), Control Small (*M* = 18.43%, 95% CI [16.73, 20.13]), Control Large (*M* = 18.92%, 95% CI [17.35, 20.49]) and Perceived Small (*M* = 19.06%, 95% CI [17.56, 20.57]) targets, however, all comparisons were non-significant (all *p*s > 0.05).

#### Number of directional changes

**FIGURE 4:**
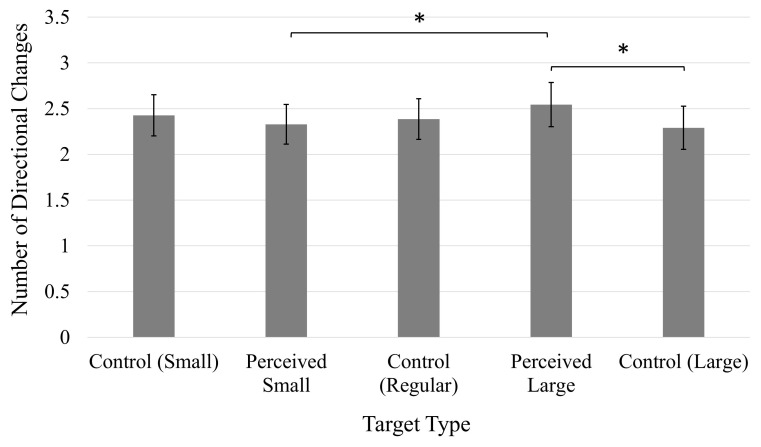
Average number of directional changes. Error bars represent 95% confidence intervals. *p < 0.05.

A significant main effect of Target, *F*(4, 196) = 3.91, *p* < 0.01, η*_*p*_*^2^ = 0.07 (Figure 4), showed that participants made significantly more directional changes when clicking the Perceived Large target in comparison to the Perceived Small and Control Large targets. There were no significant differences in the number of directional changes between any of the other target types. An increased number of directional changes were also made when the target was positioned on the right side of the screen (*M* = 2.51, 95% CI [2.29, 2.73]) compared to when positioned on the left side of the screen (*M* = 2.28, 95% CI [2.05, 2.51]), as indicated by a significant main effect of Position, *F*(1, 49) = 8.54, *p* < 0.01), η*_*p*_*^2^ = 0.15.

### Experiment 2

#### Excluded data

In total, 2.16% of all trials were excluded from analysis in Experiment 2 (early cursor movement during the 200 ms mask: 1.64%, unusable cursor/timestamp data: 0.38%, trial duration longer than 5,000 ms: 0.10%, click-point outside target boundaries: 0.04%).

#### Perceptual comparisons

Participants’ perceptual comparison scores are provided in Table 6. As in Experiment 1, participants’ perceptual comparison scores indicated the illusory context successfully influenced participants’ perceptions of target size, except for the Perceived Large (Far) target, which once again had comparatively low scores. Again, the illusion appeared to have an exaggerated effect in a small subset of responses.

**TABLE 6:** Experiment 2 perceptual comparisons.

Onscreen position		Right					
		Control Small	Perceived Small	Control	Perceived Large (Far)	Perceived Large	Control Large
**Left**	**Control Small**	–	84%*	–	96%	96%	–
	**Perceived Small**	72%*	–	**84%**	**82%**	**94%**	98%
	**Control**	–	**92%**	–	**44%**	**78%**	–
	**Perceived Large (Far)**	98%	**96%**	**44%**	–	**76%**	90%
	**Perceived Large**	98%	**96%**	**88%**	**80%**	–	82%*
	**Control Large**	–	100%	–	96%	82%*	–

Scores represent the percent of comparisons that demonstrated the expected size ordering (Smallest to Largest): Control (Small) < Perceived Small < Control < Perceived Large (Far) < Perceived Large < Control Large. **Bolded scores represent the comparisons between the same-sized targets.** Comparisons between Control stimuli (all 100%) not included. *The fact that this value is less than 100% suggests that on the remaining percent of trials participants reported the illusory target as smaller or larger than the veridically smaller (Control Small) or larger (Control Large) targets respectively, suggesting the presence of an exaggerated illusory effect.

#### Click-point accuracy

As in Experiment 1, participants’ average accuracy scores violated the assumption of normality (distributions ranged from moderately to severely positively skewed on a consistent basis). A log transformation was applied to the data, and the values reported here have been back-transformed into the original units (px). A significant main effect of Time, *F*(1, 49) = 33.47, *p* < 0.001, η*_*p*_*^2^ = 0.41, showed that participants were less accurate in the second block of trials following the manipulation (*M* = 6.40 px, 95% CI [5.15, 7.96]) compared to the first, pre-manipulation block (*M* = 4.48 px, 95% CI [3.64, 5.50]). A significant main effect of Position, *F*(1, 49) = 6.92, *p* < 0.05, η*_*p*_*^2^ = 0.12, also indicated that participants were more accurate when the target was presented on the right side of the screen (*M* = 5.21 px, 95% CI [4.23, 6.44]) than when presented on the left (*M* = 5.50 px, 95% CI [4.51, 6.68]).

**FIGURE 5:**
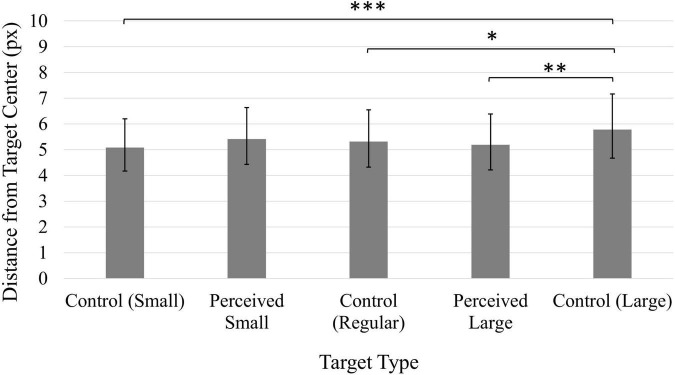
Average distance from click position to target center. Values have been back-transformed into original measurement value (px). Smaller values indicate higher accuracy. Error bars represent 95% confidence intervals. *p < 0.01, ^**^p < 0.01, ^***^p < 0.001.

Finally, a main effect of Target, *F*(4, 196) = 6.92, *p* < 0.001, η*_*p*_*^2^ = 0.12 (Figure 5), was also found to be significant. Accuracy was generally worse when participants clicked on the Control Large target. There were no significant comparisons amongst the same-sized targets (Perceived Small, Perceived Large, and Control Regular).

#### Movement time

**FIGURE 6:**
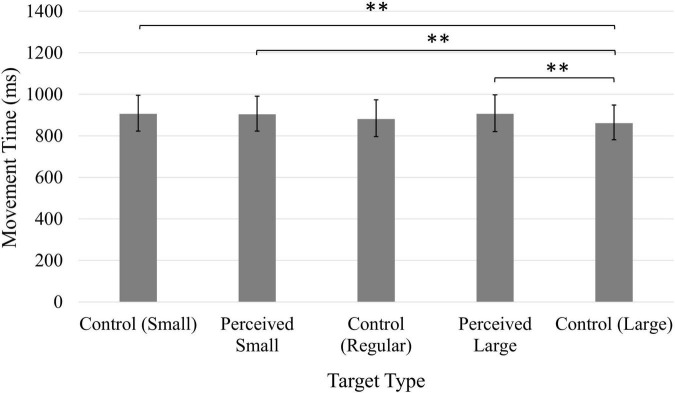
Average movement time (ms). Error bars represent 95% confidence intervals. ^**^p < 0.01.

Participants’ average movement time scores were consistently non-normal (moderately to severely positively skewed), and a log transformation was applied to the data. The values reported here have been back-transformed into the original units (ms). Participants were significantly faster during the second block of trials following the experimental manipulation (*M* = 820.35 ms, 95% CI [741.31, 905.73]) compared to the first block of trials (*M* = 968.28 ms, 95% CI [874.98, 1069.06]), as confirmed by a significant main effect of Time, *F*(1, 49) = 32.99, *p* < 0.001, η*_*p*_*^2^ = 0.40. A main effect of Position, *F*(1, 49) = 9.74, *p* < 0.01, η*_*p*_*^2^ = 0.17, was also significant, and participants were faster when the target was presented on the left side of the screen (*M* = 877.00 ms, 95% CI [796.16, 968.28]) compared to targets presented on the right (*M* = 903.65 ms, 95% CI [822.25, 993.12]). A significant main effect of Target, *F*(4, 196) = 6.63, *p* < 0.001, η*_*p*_*^2^ = 0.12 (Figure 6), indicated the decreased accuracy observed when clicking on the Control Large target was also associated with shorter movement times; participants were faster when clicking on the Control Large target compared to the Control Small, Perceived Small, and Perceived Large targets.

#### Deceleration phase

Deceleration phases were significantly longer during the first block of trials (*M* = 86.56% of total movement, 95% CI [84.80, 88.314]) compared to the second block of trials (*M* = 83.50% of total movement, 95% CI [81.60, 85.40]), as indicated by a significant main effect of Time, *F*(1, 49) = 31.07, *p* < 0.001, η*_*p*_*^2^ = 0.39. A significant main effect of Target, *F*(2, 196) = 3.81, *p* < 0.01, η*_*p*_*^2^ = 0.07, indicated longer deceleration phases when clicking on the Perceived Small target (*M* = 85.95%, 95% CI [84.16, 87.73]) compared to the Control Large target (*M* = 84.02%, 95% CI [81.98, 86.07]; *p* < 0.05). There were no other significant comparisons involving the Control Small (*M* = 84.68%, 95% CI [82.80, 86.56]), Control Regular (*M* = 85.16%, 95% CI [83.51, 86.81]) and Perceived Large (*M* = 85.33%, 95% CI [83.38, 87.28]) targets (all *p*s > 0.05).

#### Area under the curve

Cursor trajectories were significantly more curved during the second block of trials, after the manipulation (*M* = 17489.86 px, 95 CI [15834.25, 19145.46]) than compared to the first block of trials (*M* = 16131.00 px, 95% CI [14476.78, 17785.23]), as confirmed by a significant main effect of Time, *F*(1, 49) = 7.08, *p* < 0.05, η*_*p*_*^2^ = 0.13. A significant main effect of Position, *F*(1, 49) = 25.07, *p* < 0.001, η*_*p*_*^2^ = 0.34, indicated that cursor trajectories were also more curved when the target was presented on the right side of the screen (*M* = 18914.69 px, 95% CI [16817.29, 21012.08]) than when presented on the left (*M* = 14706.17 px, 95% CI [13299.45, 16112.90]).

#### Time of maximum deviation

A significant Time × Position interaction, *F*(1, 49) = 6.759, *p* < 0.05, η*_*p*_*^2^ = 0.12, indicated that while the maximum deviation occurred earlier during the first block of trials for both leftward presented targets (Pre Manipulation: *M* = 20.43% of movement time, 95% CI [18.46, 22.39], Post Manipulation: *M* = 23.27%, 95% CI [21.30, 25.23], *p* < 0.001) and rightward presented targets (Pre Manipulation: *M* = 18.58%, 95% CI [16.67, 20.49], Post Manipulation: *M* = 22.93%, 95% CI [20.87, 24.99], *p* < 0.001), differences in the time of maximum deviation between left- and rightward presented targets only occurred during the first block of trials, during which the maximum deviation occurred earlier for rightward positioned targets (*p* < 0.001) than rightward positioned targets. There was no significant difference in the time at which the maximum deviation occurred between left- and rightward presented targets in the second block of trials (*p* > 0.05).

#### Number of directional changes

**FIGURE 7:**
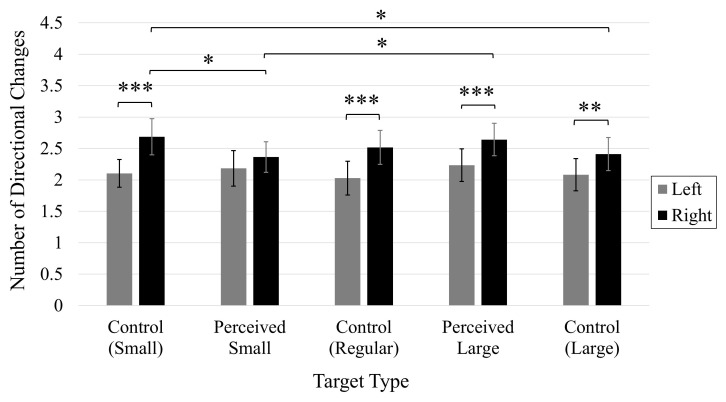
Average number of directional changes. Error bars represent 95% confidence intervals. *p < 0.05, ^**^p < 0.01, ^***^p < 0.001.

A significant Position × Stimuli interaction, *F*(4, 196) = 2.76, *p* < 0.05, η*_*p*_*^2^ = 0.05 (Figure 7), indicated that an increased number of directional changes were made when clicking on each target type when positioned on the right side of the screen compared to when presented on the left side, except for the Perceived Small target, for which the number of directional changes did not significantly differ between target positions (*p* > 0.05). There were no significant differences in the number of directional changes made by participants when clicking on targets presented on the left side of the screen (all *p*s > 0.05). However, significantly more corrections were made when clicking on the Control Small target in comparison to the Perceived Small target, and the Control Large target when these targets were presented on the right side of the screen. Cursor movements toward the Perceived Large target also involved more corrections in comparison to the Perceived Small target when presented on the right side of the screen. The fact that these comparisons were only significant when the targets were presented on the right side of the screen suggests the effect of the illusion may have been highlighted by the increased difficulty associated with a rightward movement on the trackpad.

### Experiment 3

#### Excluded data

In total, 3.14% of all trials were excluded from analysis in Experiment 3 (early cursor movement during the 200 ms mask: 2.60%, unusable cursor/timestamp data: 0.10%, trial duration longer than 5,000 ms: 0.44%).

#### Perceptual comparisons

Participants’ perceptual comparison scores are provided in Table 7. As in Experiments 1 and 2, the illusory context successfully influenced participants’ perceptions of target size, however, this was not the case for the Perceived Large (Far) target. An exaggerated influence of the illusion was observed in a small portion of participants’ responses, regardless of the target’s on-screen position.

**TABLE 7:** Experiment 3 perceptual comparisons.

Onscreen position		Right					
		Control Small	Perceived Small	Control	Perceived Large (Far)	Perceived Large	Control Large
**Left**	**Control Small**	–	80%*	–	100%	100%	–
	**Perceived Small**	76%*	–	**88%**	**86%**	**98%**	96%
	**Control**	–	**84%**	–	**46%**	**82%**	–
	**Perceived Large (Far)**	98%	**94%**	**58%**	–	**80%**	92%
	**Perceived Large**	100%	**92%**	**76%**	**76%**	–	86%*
	**Control Large**	–	94%	–	94%	84%*	–

Scores represent the percent of comparisons that demonstrated the expected size ordering (Smallest to Largest): Control (Small) < Perceived Small < Control < Perceived Large (Far) < Perceived Large < Control Large. **Bolded scores represent the comparisons between the same-sized targets.** Comparisons between Control stimuli (all 100%) not included. *The fact that this value is less than 100% suggests that on the remaining percent of trials, participants reported the illusory target as smaller or larger than the veridically smaller (Control Small) or larger (Control Large) targets respectively, suggesting the presence of an exaggerated illusory effect.

#### Click-point accuracy

**FIGURE 8:**
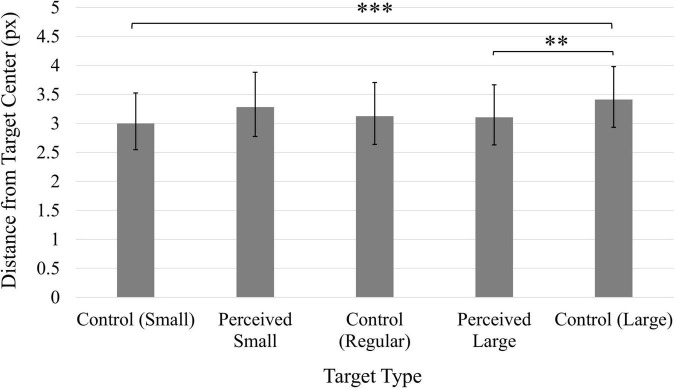
Average distance from click position to target center. Values have been back-transformed into original measurement value (px). Smaller values indicate higher accuracy. Error bars represent 95% confidence intervals. ^**^p < 0.01, ^***^p < 0.001.

As was the case for Experiments 1 and 2, participants’ average accuracy scores were consistently positively skewed, and a log transformation was applied to the data. The values reported here have been back-transformed into the original units (px). A significant main effect of Time, *F*(1, 49) = 42.44, *p* < 0.001, η*_*p*_*^2^ = 0.46, indicated that participants’ accuracy increased following the manipulation (*M* = 2.37, 95% CI [2.05, 2.74]) compared to before the manipulation (*M* = 4.27, 95% CI [3.44, 5.29]). A significant main effect of Target, *F*(4, 196) = 5.66, *p* < 0.001, η*_*p*_*^2^ = 0.10 (Figure 8), indicated the Control Large target generated significantly worse accuracy in comparison to the Control Small and Perceived Large targets. All other comparisons were non-significant (*p*s > 0.05).

#### Movement time

A significant main effect of Time, *F*(1, 49) = 61.47, *p* < 0.001, η*_*p*_*^2^ = 0.56, indicated that participants were slower following the manipulation (*M* = 1358.33 ms, 95% CI [1246.81, 1469.86] compared to before the manipulation (*M* = 1059.90 ms, 95% CI [951.28, 1168.52]).

#### Deceleration phase

Deceleration phases were significantly longer in the second block of trials, following the manipulation (*M* = 89.03%, 95% CI [87.64, 90.43]) compared to the first block of trials (*M* = 85.86%, 95% CI [83.99, 87.73]), as indicated by a significant main effect of Time, *F*(1, 49) = 22.45, *p* < 0.001, η*_*p*_*^2^ = 0.31. A significant main effect of Target was also observed, *F*(4, 196) = 3.298, *p* < 0.05, η*_*p*_*^2^ = 0.06, however there were no significant comparisons between the different target types (all *p*s > 0.05); Control Small (*M* = 87.14%, 95% CI [85.56, 88.72]), Perceived Small (*M* = 87.88%, 95% CI [86.24, 89.53]), Control Regular (*M* = 87.09%, 95% CI [85.25, 88.92]), Perceived Large (*M* = 88.35%, 95% CI [86.88, 89.82]), Control Large (*M* = 86.77%, 95% CI [85.12, 88.41]).

#### Area under the curve

**FIGURE 9:**
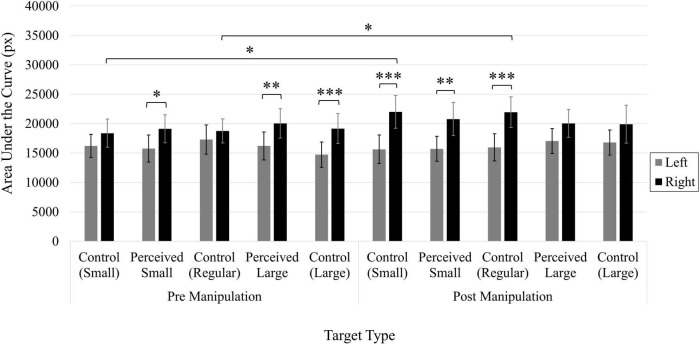
Average area under the curve (px). Error bars represent 95% confidence intervals. ^**^p < 0.01, ^***^p < 0.001.

A three-way Time × Position × Target interaction was shown to be significant, *F*(4, 196) = 2.85, *p* < 0.05, η*_*p*_*^2^ = 0.06 (Figure 9). Prior to the manipulation, trajectories were more curved when the Perceived Small, Perceived Large, and Control Large targets were presented on the right side of the screen compared to the left side; the position of the target had no influence on the Control Small and Control Regular targets (*p*s > 0.05). After the manipulation, trajectories were more curved when the Control Small, Perceived Small, and Control Regular targets were presented on the right side of the screen compared to the left side; the position of the target had no influence on the Perceived Large and Control Large targets (*p*s > 0.05). When clicking on Control Small and Control Regular targets presented on the right side of the screen, trajectory curvature increased following the manipulation. Otherwise, the manipulation did not influence trajectory curvature (*p*s > 0.05). There were no significant comparisons between any of the target types on either the left or right side of the screen, or before or after the manipulation (*p*s > 0.05).

#### Time of maximum deviation

A significant main effect of Time, *F*(1, 49) = 30.03, *p* < 0.001, η*_*p*_*^2^ = 0.38 indicated the maximum deviation occurred earlier in the second block of trials, following the manipulation (*M* = 15.41% of the total movement, 95% CI [13.89, 16.92]) compared to the first block of trials (*M* = 19.71%, 95% CI [17.69, 21.74]).

#### Number of directional changes

**FIGURE 10:**
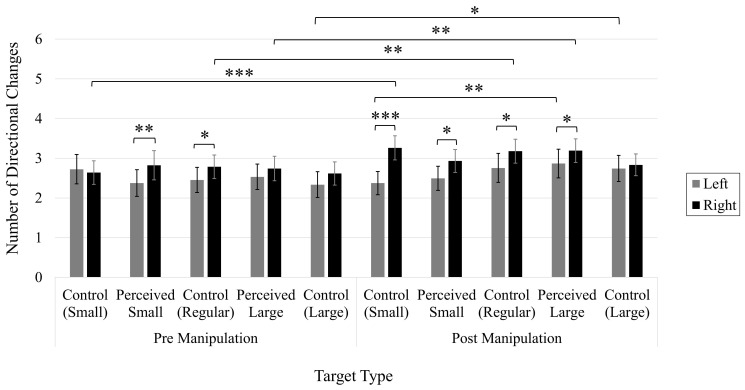
Average number of directional changes. Error bars represent 95% confidence intervals. *p < 0.05, ^**^p < 0.01, ^***^p < 0.001.

A significant three-way Time × Position × Target interaction was observed, *F*(4, 196) = 4.75, *p* < 0.01, η*_*p*_*^2^ = 0.09 (Figure 10). Prior to the manipulation, participants made significantly more directional changes when clicking on rightward positioned Perceived Small and Control Regular targets in comparison to when these targets were presented on the left side of the screen. After the manipulation, more directional changes were observed when each target was presented on the right side of the screen compared to the left side, with the exception of the Control Large target, for which the number of directional changes did not differ between onscreen positions (*p* > 0.05). The number of directional changes increased following the manipulation when clicking on rightward positioned Control Small, Control Regular, and Perceived Large targets. The number of directional changes also increased post-manipulation when clicking on Control Large targets presented on the left side of the screen. Prior to the manipulation, the number of directional changes did not significantly differ between target types (all *p*s < 0.05). Following the manipulation, however, participants made significantly more directional changes when clicking on the Control Small target compared to the Perceived Large target when these targets were presented on the left side of the screen. Otherwise, there were no significant comparisons between the different targets on either the left or right side of the screen, or before or after the manipulation (*p*s > 0.05).

## General discussion

The goal of this study was to test the feasibility of using remote data collection methods to study the type of visually guided actions typically measured within the laboratory setting. Remote data collection provides several potential advantages for behavioral research, including the opportunity to measure a large sample size in a short period of time, as well as presenting a cost-effective and convenient option for both the experimenter and the participant, who can participate in the comfort of their own home. The methods outlined in this study present a novel approach to the collection and analysis of visually guided cursor movement and present an example of how these measures can be applied to investigations of visual perception and action.

Participants’ perceptual comparison scores consistently indicated that the presence of the illusion successfully influenced the perceived size of the targets. However, all three experiments failed to demonstrate an influence of perceived target size on click-point accuracy or movement time. In this sense, these results seem to provide evidence in favor of a visually guided action system that operates separate from the influence of perception, at least in the context of the visuomotor transformation used in this study (i.e., transformation of the proximal digit movement to the distal cursor movement). The results of the current study are similar to those of a study conducted by Janczyk et al. (2013) in which participants’ perceptual judgments were influenced by irrelevant stimulus dimensions during a Garner-interference speed classification task, while cursor movements directed toward these stimuli were unaffected. Thus, there appears to be increasing evidence that the cursor movements are unaffected by perceptual intrusions.

While the illusory context did not appear to influence participants’ click-point accuracy or movement time as expected, these results suggest the illusory context did influence the trajectories of participants’ cursor movements toward the target. More directional changes were observed when clicking the Perceived Large target in comparison to the Perceived Small target in Experiments 1 and 2. In general, cursor movements directed toward rightward positioned targets demonstrated larger AUCs (more curved trajectories) and more directional changes, suggesting participants may have had more difficulty with rightward movements on the trackpad compared to leftward movements. However, the Perceived Small target was the only target type to not produce an increase in the number of directional changes when positioned on the right compared to the left side of the screen in Experiment 2. This left-right differentiation was observed in the trajectory curvature and number of directional changes toward the Perceived Small target in Experiment 3, however, the increased emphasis on accuracy in this experiment did not influence the Perceived Small target as it did the other target types. Taken together, these results suggest participants’ cursor paths were not influenced to the same degree by the on-screen position of the target or the demands of the task when clicking on the Perceived Small target in comparison to the other target types.

Whereas the perception-action model predicts participants’ cursor paths will be unaffected by the illusory context, and therefore should remain similar regardless of the type of target, a planning-control model (e.g., Glover and Dixon, 2002; Glover, 2004) might predict the number of corrections to vary as a result of the illusory context, as was observed in this study. Specifically, if the illusory context decreased the perceived size of the target (Perceived Small target) during the planning stage, the control stage of that movement may require less corrections than that of a movement that was planned toward a target perceived to be larger than its physical size (Perceived Large target). This could potentially explain the increased number of corrections and resulting increased accuracy and movement time observed when clicking the Perceived Large target. Further, if cursor movements toward the Perceived Small target benefited from a more accurate planning stage, these movements would likely be less influenced by the present task demands (i.e., target position or speed and accuracy of the movement) than less accurately planned movements, as observed in Experiments 2 and 3. Further evidence in favor of this explanation comes from the time at which the maximum deviation from the idealized trajectory occurred. A significant effect of Target Type in Experiment 1 suggested the maximum deviation occurred earliest when clicking the Perceived Large target, and latest when clicking the Perceived Small target. The early deviations in response to the Perceived Large target may represent a less accurate planning stage and thus explain why more corrective movements were required when clicking this type of target compared to the Perceived Small target, for which the maximum deviation occurred later, possibly indicating a more accurate planning stage. However, despite the significant main effect, the more stringent Bonferroni corrected pairwise comparisons between the different target types did not reach statistical significance.

A planning-control model of this nature such as the one proposed by Glover and Dixon (2002) and Glover (2004) is not without its own share of criticisms, however (Danckert et al., 2002; Franz, 2003; Goodale and Milner, 2004; Handlovsky et al., 2004; Westwood, 2004; Franz et al., 2005). Nevertheless, the results of this study appear to suggest the planning of these cursor movements may have been influenced by the illusory context, while end-point measures such as final accuracy and movement time were not. As such, the results of the current study may be explained by both the perception-action and planning-control models of visually guided action in the control of onscreen cursor movements.

Alternatively, it is also possible that participants’ cursor movements were not influenced by the effect of the illusion, but rather these differences were produced by the position of the context circles, and their varying proximities to the target circle. This proposal is similar to the one used previously to explain the results of studies observing differences in grip aperture when grasping; participants may treat pictorially presented context circles as obstacles or distractors when grasping a central disk or “chip” and respond by adjusting their grip apertures (Haffenden and Goodale, 2000; Haffenden et al., 2001). In this study, the context circles comprising the Perceived Large target may have been more likely to be treated as “obstacles” or “distractors” due to their close proximity to the target, in comparison to the farther positioned context circles of the Perceived Small target. If participants were treating the context circles as obstacles, they may have generated more directional changes when clicking the Perceived Large target to avoid a “collision” between the cursor and context circles. It seems unlikely, however, that the context circles in the current experiment would elicit such obstacle avoidance mechanisms, as there was no associated risk of collision, and participants were not given any instruction to avoid the context circles. An alternative possibility is that the proximity of the context circles surrounding the Perceived Large target may have provided participants with a larger “general area” (i.e., combining the target and context circles) to which initial cursor movements were directed to. Additional directional changes would therefore be required during later stages of the movement, once the center target is considered separate from the entire configuration. Unfortunately, removal of the Perceived Large (Far) target from the analysis meant we could not distinguish the illusory effect induced by the context circles and their proximity to the target.

Participants demonstrated shorter deceleration phases when clicking on the Control Large target in comparison to the Perceived Small target in Experiment 2, and a significant effect of Target Type in Experiment 3 suggested deceleration phases were shortest in response to the Control Large target (however, Experiment 3 pairwise comparisons did not reach statistical significance). Observing shorter deceleration phases in response to the veridically larger Control Large target makes intuitive sense, as the larger target requires less precision at later stages of the movement. These results support other research demonstrating shorter deceleration phases toward targets perceived as larger (Handlovsky et al., 2004). However, it is less clear why the Perceived Small target in particular would generate longer deceleration phases in comparison. While it would be reasonable to expect longer deceleration phases for targets perceived as smaller, and therefore requiring increased precision, this was not the case for the veridically smaller Control Small target. If cursor movements directed toward the Perceived Small target benefited from a more accurate planning phase as suggested above, then in fact we might expect shorter deceleration phases when clicking on this target type, rather than the longer deceleration phases observed in Experiment 2. It should be noted, however, that cursor movements such as those generated in this experiment have the potential to be much more sporadic and unorganized than the types of reaching-to-grasp or pointing movements typically investigated (see Figure 3 for an example of the variability of movement trajectories between trials). As a result, the deceleration phase (as defined in this study as the proportion of the movement following peak velocity) of a cursor movement may not be as reliable an indicator of online control or an increase in precision as it is when considering visually guided hand movements.

The results of this experiment also provide valuable information about how the target’s onscreen position influenced participants’ cursor movement. In general, participants cursor movements were slower, more curved, and consisted of a higher number of corrective movements when the target was presented on the right side of the screen than compared to when presented on the left. The time at which the maximum deviation from the idealized trajectory path occurred differed between left and right targets in the first block of trials in Experiment 2. Following the manipulation, however (increased emphasis on the speed at which the task was performed), this difference disappeared. The fact that these effects were observed regardless of the type of target being presented suggest these changes in the speed and trajectory of the cursor movements toward leftward and rightward presented targets are more likely a result of all participants controlling the cursor with their right hand, rather than the result of the illusory context.

Whereas, ipsilateral reaching movements are typically faster and more accurate than contralateral movements (Carson et al., 1993; Hodges et al., 1997) the additional mechanical constraints present when using the right hand to perform a rightward cursor movement on a trackpad introduces certain difficulties which may have contributed to the effects observed in this study. For example, using the right hand to perform a leftward movement of the cursor simply requires the extension of the digit on the touchpad, while a rightward movement requires adduction of the index finger (or rightward abduction of the middle finger), as well as a necessary adduction of the wrist. These results suggest future investigations of trackpad-controlled cursor movement and human-computer interaction in general should consider the added mechanical constraints associated with a rightward compared to a leftward cursor movement.

### Methodological considerations

There are several methodological considerations that may have also contributed to the absence of an observed effect of the illusion on the performance variables in this study. First, the task itself was relatively easy, simply requiring participants to move their cursors toward the target and click the center quickly and accurately. Despite encouraging participants to perform the task faster in Experiment 2 and more accurately in Experiment 3, the actual task requirements did not effectively change in these experiments. Additionally, while the type of target varied between each trial, the target only ever appeared at one of two onscreen positions, on either the left or right side. This meant that regardless of the type of target presented, the center of the target was always located at the same leftward or rightward position. Therefore, even if the perceived size of the target did affect participants’ performance of the task, the simple, repetitive nature of the task and participants’ overall high performance may have masked the influence of the illusion.

Second, participants performed the task in a closed-loop fashion and visual feedback of the target was always available. Participants therefore had ample opportunity to refine and adjust their movements online to achieve a consistently high level of accuracy. Previous research successfully demonstrating an influence of the Ebbinghaus illusion on visually guided aiming movements has typically involved removal of visual feedback to some degree, either by removing vision of the hand (van Donkelaar, 1999), or the target (Fischer, 2001; Alphonsa et al., 2016). Both the perception-action and planning-control models predict that by removing visual feedback of the target, a greater emphasis will be placed on participants’ sensorimotor memory of the target’s position, therefore recruiting the perceptual system’s involvement in the task, and increasing the likelihood of an illusory influence. According to the perception-action model, the action toward the target’s remembered location will rely primarily on stored representations of the target within the perceptually dominated ventral stream and will therefore be more susceptible to the original illusory context of the target. Similarly, the planning-control model suggests that without visual feedback of the target facilitating the online corrections occurring during the action’s “control” phase, the movement will be primarily guided by the representation of the target’s position constructed during the “planning” phase, which is also susceptible to the illusory context prior to effector movement.

Third, the perceptual comparison task used in this study to confirm if the illusions effectively induced a change in perceived target size may have influenced the strength of the illusion differently for the perceptual task compared to the movement task. For example, this study utilized a forced choice task between two target stimuli. It may be argued that the division of attention required for this type of task may be more likely to produce an illusory effect (Pavani et al., 1999; Franz et al., 2000; Foster and Franz, 2014), while the directed focus during an action toward a single target may reduce the influence of the illusion. It is therefore a possibility that the illusory stimuli used in this study were more effective during the perceptual comparison task than during the experimental trials, which could also explain the lack of any observed influence of the illusion when acting on the single target. Conversely, as participants were not constrained regarding the amount of time required to perform the perceptual comparisons, it is possible the potential for increased inspection time may have in fact weakened the effect of the illusion for those who spent more time viewing the targets (Bressan and Kramer, 2021).

Finally, the online nature of this experiment involved participants performing the experimental task remotely, using a wide range of device types, screen sizes and resolutions, and without experimenter supervision. As such, the presentation of the stimuli likely varied to some degree depending on the screen size and resolution of the device used by participants to complete the experiment. For example, the container in which the onscreen display was presented was set as relatively small (800 × 600 px) to accommodate the presentation of the experiment on a wide variety of screen sizes. The strength of the Ebbinghaus illusion has shown to increase as the size of the stimuli increase (Massaro and Anderson, 1971; Knol et al., 2015) and therefore the smaller display may have weakened the influence of the illusion. Each experiment was conducted using a within-subjects repeated measures design as an attempt to control for the variety of display presentations between participants. However, the minimized experimental control inherent in this form of remote data collection remains a possible threat to the internal validity of the experiment.

## Conclusion

Here we have provided an example of how remote data collection methods may be used to conduct behavioral research outside of the laboratory setting. Using the Ebbinghaus illusion, participants’ perceptions of the onscreen stimuli were influenced during a point-and-click task performed remotely using their own laptop devices. While the trajectory of the cursor movement appeared to be influenced by the perceived size of the target illusory context, end-point measures such as click-point accuracy and movement time were not influenced by the illusory context. Despite the convenience provided by remote data collection, those utilizing remote data collection should carefully consider the appropriate methodological considerations and anticipate the potential decrease in experimental control associated with conducting behavioral research outside of the laboratory environment.

## Data availability statement

The original contributions presented in this study are publicly available. This data can be found here: doi: 10.34990/FK2/NC0MPM.

## Ethics statement

The studies involving human participants were reviewed and approved by the Psychology/Sociology Research Ethics Board (P/SREB) at the University of Manitoba. Written informed consent to participate in this study was provided by the participants.

## Author contributions

RL was responsible for construction of the online experiment, as well as collection and analysis of the data, and the writing of the original manuscript draft. JM provided funding acquisition, project resources, and supervision. Both authors contributed to the conception and design of the study, as well as revision of the submitted manuscript.

## Footnotes

1
https://github.com

2
https://firebase.google.com
