# Corrigendum: Use of remote data collection methodology to test for an illusory effect on visually guided cursor movements

**DOI:** 10.3389/fpsyg.2022.1042774

**Published:** 2022-11-15

**Authors:** Ryan W. Langridge, Jonathan J. Marotta

**Affiliations:** Perception and Action Lab, Department of Psychology, University of Manitoba, Winnipeg, MB, Canada

**Keywords:** Ebbinghaus illusion, Titchener circles, cursor control, perception, action

In the published article, there was an error in Figure 4 as published. The Y-Axis Label incorrectly stated: Number of Corrective Movements. The correct Y-Axis Label is: Number of Directional Changes. The corrected Figure 4 and its caption appear below.

**Figure 4:**
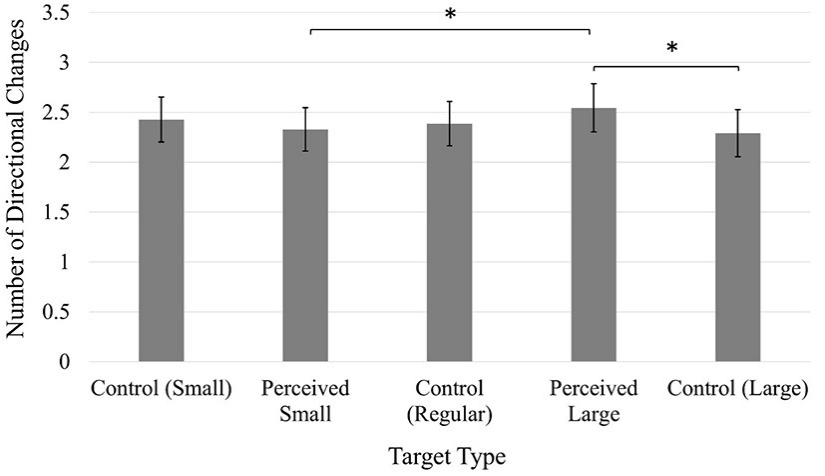
Average number of directional changes. Error bars represent 95% confidence intervals. *p < 0.05.

In the published article, there was an error in Figure 7 as published. The Y-Axis Label incorrectly stated: Number of Corrective Movements. The correct Y-Axis Label is: Number of Directional Changes. The corrected Figure 7 and its caption appear below.

**Figure 7:**
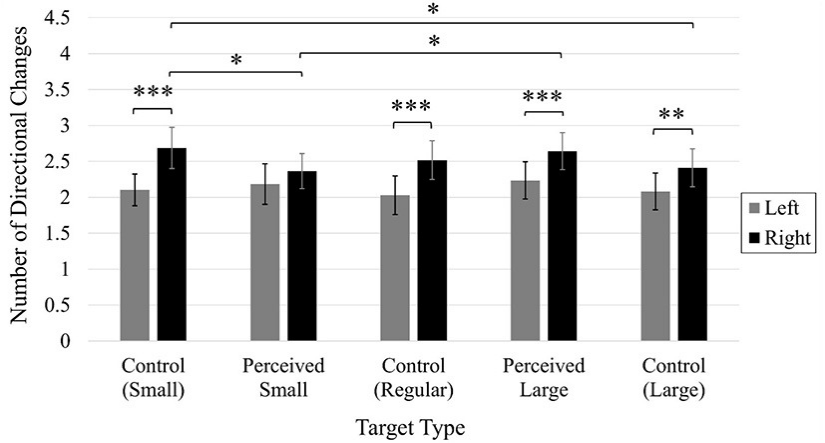
Average number of directional changes. Error bars represent 95% confidence intervals. *p < 0.05, **p < 0.01, ***p < 0.001.

In the published article, there was an error in Figure 10 as published. The Y-Axis Label incorrectly stated: Number of Corrective Movements. The correct Y-Axis Label is: Number of Directional Changes. The corrected Figure 10 and its caption appear below.

**Figure 10:**
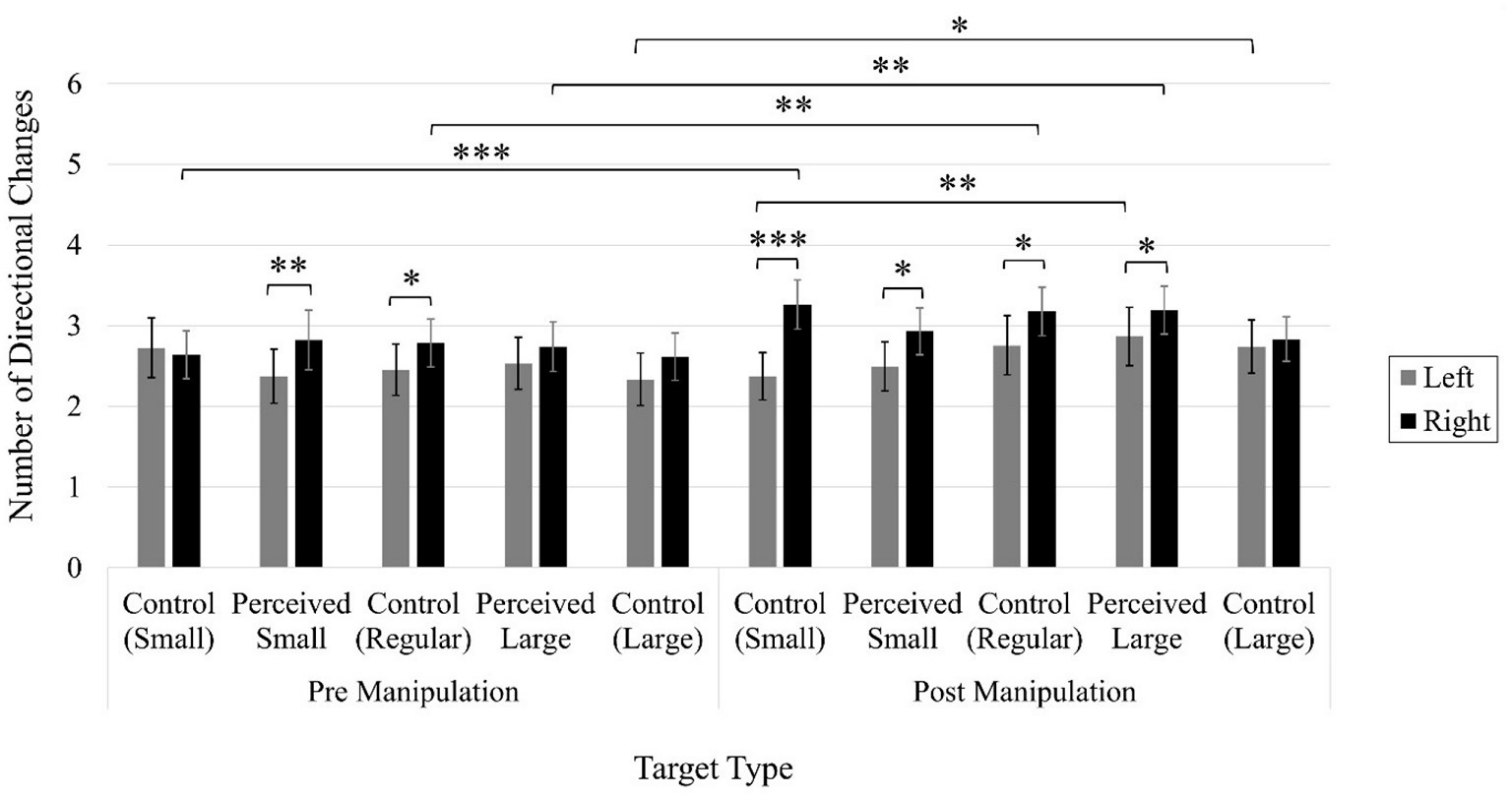
Average number of directional changes. Error bars represent 95% confidence intervals. *p < 0.05, **p< 0.01, ***p< 0.001.

The authors apologize for these errors and state that they do not change the scientific conclusions of the article in any way. The original article has been updated.

## Publisher's note

All claims expressed in this article are solely those of the authors and do not necessarily represent those of their affiliated organizations, or those of the publisher, the editors and the reviewers. Any product that may be evaluated in this article, or claim that may be made by its manufacturer, is not guaranteed or endorsed by the publisher.

